# Antibiotic Susceptibility, Resistance Gene Determinants and Corresponding Genomic Regions in *Lactobacillus amylovorus* Isolates Derived from Wild Boars and Domestic Pigs

**DOI:** 10.3390/microorganisms11010103

**Published:** 2022-12-30

**Authors:** Monika Moravkova, Iveta Kostovova, Katerina Kavanova, Radko Pechar, Stanislav Stanek, Ales Brychta, Michal Zeman, Tereza Kubasova

**Affiliations:** 1 Department of Microbiology and Antimicrobial Resistance, Veterinary Research Institute, Hudcova 296/70, 621 00 Brno, Czech Republic; 2 Department of Experimental Biology, Faculty of Science, Masaryk University, Kamenice 753/5, 625 00 Brno, Czech Republic; 3 Food Research Institute Prague, Radiová 1285/7, 102 00 Prague, Czech Republic; 4 Department of Microbiology, Nutrition and Dietetics, Faculty of Agrobiology, Food and Natural Resources, Czech University of Life Sciences Prague, Kamýcká 129, 165 00 Prague, Czech Republic; 5 MIKROP ČEBÍN a.s., Čebín 416, 664 23 Čebín, Czech Republic

**Keywords:** antibiotic resistance, *tetW*, *ermB*, *Lactobacillus amylovorus*, domestic pigs, wild boars

## Abstract

Restrictions on the use of antibiotics in pigs lead to the continuous search for new probiotics serving as an alternative to antibiotics. One of the key parameters for probiotic bacteria selection is the absence of horizontally transmissible resistance genes. The aim of our study was to determine antibiotic susceptibility profiles in 28 *Lactobacillus amylovorus* isolates derived from the digestive tract of wild boars and farm pigs by means of the broth microdilution method and whole genome sequencing (WGS). We revealed genetic resistance determinants and examined sequences flanking resistance genes in these strains. Our findings indicate that *L. amylovorus* strains from domestic pigs are predominantly resistant to tetracycline, erythromycin and ampicillin. WGS analysis of horizontally transmissible genes revealed only three genetic determinants (*tetW*, *ermB* and *aadE*) of which all *tetW* and *ermB* genes were present only in strains derived from domestic pigs. Sequence analysis of coding sequences (CDS) in the neighborhood of the *tetW* gene revealed the presence of site-specific recombinase (*xerC*/*D*), site-specific DNA recombinase (*spoIVCA*) or DNA-binding transcriptional regulator (*xre*), usually directly downstream of the *tetW* gene. In the case of *ermB*, CDS for omega transcriptional repressor or mobilization protein were detected upstream of the *ermB* gene.

## 1. Introduction

Currently, the fight against the emergence of multidrug-resistant bacteria represents one of the key priorities of the healthcare system. One of the important factors involved in the spread of antibiotic resistance is the excessive and improper use of antibiotics in food-producing animals, particularly poultry, pigs and cattle. Tetracyclines and penicillins are generally the most commonly used in food-producing animals, accounting for 30% and 27% of sales, respectively. High consumption is also observed for sulfonamides, macrolides and lincosamides [1]. Similarly, tetracyclines and penicillins are the classes most commonly used in pig production. The application of macrolides in pigs, as the member of the group of critically important antibiotics with the highest priority in humans, significantly varies between countries. The most common indication of antibiotic application in pigs are gastrointestinal and respiratory infections. These infections are commonly treated by tetracycline, lincosamides, macrolides, colistin, tylosin, pleuromutilins and amoxicillin [2]. The monitoring of resistant pathogenic and indicator bacteria in pigs from different countries revealed an association between antimicrobial use and an increased number of resistant bacterial strains and horizontally acquired resistance genes, particularly to tetracycline and macrolide. Variability in the occurrence of antibiotic genes in the bacterial population may vary significantly between individual countries [3,4].

In the last decade, there has been growing interest in the application of probiotics as a feed additive for farm animals. This trend results from the need to find alternative approaches to antibiotics, whose application is being significantly restricted in animals. Furthermore, probiotics have been proven to have the ability to alter the gut micro-biota beneficially and thereby improve animal health and productivity [5]. Lactobacilli are the most widely used probiotics in humans as well as animals. In addition to their beneficial properties, they are also considered safe organisms included in the list of qualified presumption of safety status developed by the European Food Safety Authority (EFSA) Scientific Committee [6]. Generally, they have the intrinsic capacity to tolerate low pH values and high bile concentrations [7].

Unfortunately, even non-pathogenic bacteria can serve as potential reservoirs of antibiotic resistance. For this reason lactobacilli and bacterial species used in the food and feed industry, for example as probiotics or starter cultures, must be tested and strains carrying transmissible resistance genes cannot be used [1]. It has been documented that lactobacilli can exhibit resistance to a number of antibiotics. Most *Lactobacillus* species are intrinsically resistant to aminoglycosides (e.g., kanamycin, streptomycin, gentamicin), glycopeptides (e.g., vancomycin) and inhibitors of the synthesis of nucleic acids (e.g., ciprofloxacin). On the other hand, they are usually susceptible to the protein synthesis inhibitors tetracycline, erythromycin, chloramphenicol and clindamycin and cell-wall synthesis inhibitors, such as ampicillin [8]. Acquired resistance in these primary susceptible strains arises either from the acquisition of resistance genes through horizontal transfer or due to the mutation of indigenous genes. Many of the antibiotic resistance genes are carried on plasmids, transposons or integrons that can act as vectors that transfer these genes to other members of the same bacterial species, as well as to bacteria of another genus or species [9]. Lactobacilli strains carrying resistance genes have been identified and isolated from a variety of dairy products and fermented foods and, in particular, from the feces or gut of humans and animals [8,10,11,12]. The transfer of resistance genes between lactobacilli and other bacterial species has been proven in a number of studies [9].

*Lactobacillus amylovorus* is a promising pig probiotic and is a characteristic representative of the swine intestinal microbiota isolated from both wild boars and domestic pigs [13]. It is a member of the *L. acidophilus* group [14]. *L. amylovorus* actively ferments starch and harbors amylolytic enzyme activity helping to increase feed digestibility. Further, *L. amylovorus* is an obligatory homofermentative and grows over a wide temperature range from 15 °C to 45 °C [15].

As in other studies, we have also isolated the species *L. amylovorus* from the GIT of domestic pigs as well as from the GIT of wild boars. Based on the available literature, we assume that the isolated strains could have a probiotic potential, and we would like to study this in future experiments. However, the first step is to determine the safety of this species as regards the potential spread of antibiotic resistance genes. Since there is only a limited number of articles dealing with the antibiotic resistance of the species *L. amylovorus*, we have decided to study its safety in greater detail to provide more information about this species isolated from pigs. In the present study, antibiotic susceptibility profiles and the presence of antibiotic resistant genes with corresponding genomic regions were determined in order to evaluate safety and produce a detailed characterization of strains of *L. amylovorus* from the gastrointestinal tract (GIT) of wild boars and domestic pigs.

## 2. Materials and Methods

### 2.1. Source of Strains, Culture and Primary Identification

The 28 strains of *L. amylovorus* used in this study were derived from culture analysis of the contents of the digestive tract of wild boars and domestic pigs. Samples of the small and large intestines were collected during wild boar hunting or at slaughterhouses in the winter season, mostly during the year 2018–2019. In total, 50 wild boars and 18 domestic pigs from 15 localities and four farms in the Czech Republic were sampled into anaerobic tubes, transferred in cooled boxes to the laboratory and subsequently cultured on Rogosa agar (Oxoid, Basingstoke, UK). Cultivation was conducted simultaneously under anaerobic (10% CO_2_/10% H_2_/80% N_2_ atmosphere in anaerobic jars with palladium catalysts; Oxoid) and microaerophilic conditions at 37 °C. Subsequent cultivation of 10 morphologically different isolates per animal was carried out on De Man, Rogosa and Sharpe agar (MRS agar; Oxoid) under anaerobic conditions at 37 °C for 48 h. Identification was performed based on sequencing analysis of the 16S rRNA gene using the primers 16S27f (AGAGTTTGATCMTGGCTCAG) and 16S1492r (TACGGYTACCTTGTTACGACTT) [16]. The resulting PCR products were purified using a QIAquick PCR Purification Kit (Qiagen, Valencia, CA, USA). The amplicons obtained by PCR were sequenced in both directions, forward and reverse, using a Mix2Seq Kit by Eurofins Genomics (Luxembourg City, Luxembourg). Isolated bacterial strains were identified based on sequence identity with reference sequences in the GenBank and EzBioCloud databases (http://www.ezbiocloud.net; accessed on 1 October 2020).

### 2.2. Antibiotic Susceptibility

Antimicrobial susceptibility was determined using broth microdilution methods according to ISO10932:2010 standards and the interpretation criteria suggested by EFSA FEEDAP Panel guidance [1,6]. The microplates were incubated for 48 h at 37 °C in an anaerobic atmosphere after which the minimal inhibition concentration (MIC) was read visually as the lowest concentration of antimicrobial substance that inhibited the growth of bacteria. The following antimicrobials were tested: ampicillin (0.125–16 mg/L), streptomycin (2–256 mg/L), tetracycline (0.5–64 mg/L), erythromycin (0.063–8 mg/L), clindamycin (0.063–8 mg/L), chloramphenicol (0.25–32 mg/L), kanamycin (0.5–2050 mg/L), gentamicin (0.125–512 mg/L), vancomycin (0.25–32 mg/L) and ciprofloxacin (0.125–128 mg/L). The accuracy of susceptibility testing was monitored by the use of quality control strains (*Lactobacillus plantarum* ATCC14917 and *Lactobacillus paracasei* ATCC334). The evaluation of susceptibility was based on microbiological cut-off values established for the *Lactobacillus acidophilus* group [6].

### 2.3. Nitrocefin Test

Isolates displaying phenotypic resistance to ampicillin were additionally tested using a nitrocefin disk (Sigma-Aldrich, Saint Louis, MO, USA). A loopful of overnight culture grown on MRS agar was smeared on the moisturized nitrocefin disk. The bacteria were considered beta-lactamase positive if a red color appeared on the strips in 15 min.

### 2.4. Whole-Genome Sequencing and De Novo Assembly

The genomic DNA of all the isolated strains was extracted using a Quick-DNA^TM^ Fecal/Soil Microbe Microprep Kit, according to the manufacturer’s instructions (Zymo Research, Irvine, CA, USA). Extracted DNA was subjected to library construction using a Nextera Library preparation kit and paired-end sequencing was performed with the NextSeq platform with a NextSeq 500/550 High Output Kit v2.5 (Illumina, Inc.; San Diego, CA, USA). Generated read sequences were trimmed with Trim Galore v.0.6.6 (www.bioinformatics.babraham.ac.uk; accessed on 1 December 2020), powered by Cutadapt v.0.6.6, which also removed low-quality reads. The quality of the remaining reads was evaluated by MultiQC v.1.9 [17]. Trimming was followed by de novo genome assembly using Unicycler v0.4.9b [18] using SPAdes v.3.14.1 [19].

### 2.5. Average Nucleotide Identity

The 16S rRNA gene sequencing did not provide definitive species identification due to the high similarity of 16S rRNA genes in closely related bacterial species. Therefore, taxonomical classification of all genomes used in this study, including those downloaded from the National Center for Biotechnology Information (NCBI) (February 2022, see Table 1), was confirmed by average nucleotide identity (ANI) calculations by FastANI v1.32 [20].

### 2.6. Genome Annotation and Comparative Genome Analysis

Genome annotation was unified for all genomes including genomes obtained from the NCBI (Table 1). Gene prediction and annotation was performed using Prokka v.1.14.6 against following databases: ISfinder, NCBI Bacterial Antimicrobial Resistance Reference Gene database and UniprotKB (SwisProt) as a part of HAMAP. All databases used by Prokka were updated as of December 2020 [21]. Protein sequences generated by Prokka were used for functional annotation based on precomputed orthology assignments using the EggNOG-mapper tool e-mapper v.2.1.6.-25-g1502c0F [22]. Protein sequences were searched against the EggNOG database (EggNogDB version 5.0.2) by the DIAMOND v.2.0.11 protein aligner [23]. Prokka generated annotation files with protein sequences were used as the input for pan-genome prediction by Roary v.3.13.0 [24] with default parameters. The insertion sequences were identified using the Prokka annotation pipeline.

### 2.7. Detection of Antibiotic Resistance Genes and Analysis of the Corresponding Genomic Regions

Horizontally acquired antibiotic resistance genes were analyzed by Abricate v.1.0.1 software (https://github.com/tseemann/abricate; accessed on 7 February 2022) with the use of the following databases: Comprehensive Antibiotic Resistance Database (CARD) [25], ResFinder [26], Argannot [27], Megares [28] and NCBI AMRFinderPlus [29]; all databases were updated on 7 February 2022. Abricate was used with parameters of minimum DNA identity of 80% and minimum sequence coverage of 80%. Artemis v.18.1.0. software was applied for the study of the corresponding genomic regions of the most commonly detected antibiotic resistance genes (*tetW* and *ermB*) [30]. Additionally, the pan-genome computed using Roary was applied to compare patterns of coding sequences (CDS) surrounding antibiotic resistance genes among all genomes. The genetic organization of sequences surrounding antibiotic resistance genes was visualized using Easyfig v.2.2.5 software [31]. Global alignments were performed by the ClustalO web server [32]. Identification and annotation of prophage sequences related to antibiotic resistance genes was performed using the PHASTER (PHAge Search Tool—Enhanced Release) web server [33].

### 2.8. Sequence Comparison of Antimicrobial Resistance Genes and Construction of a Phylogenetic Tree

*tetW* genes from strains used in our study and *tetW* genes retrieved from *L. amylovorus* genomes downloaded from NCBI were compared using MEGA-X software [34] and NCBI blastn [35] to determine and show the relationships among the *tetW* antibiotic resistance genes in *L. amylovorus* strains. Phylogenetic trees were constructed based on the Maximum Likelihood Method and Tamura 3-parameter model [36] using the MEGA-X software evaluated by 1000 bootstrap replication [34].

### 2.9. Prediction of Plasmid Contigs

All contigs were analyzed by Abricate using the PlasmidFinder database downloaded on 7 February 2022 [37] and by Platon v.1.6 software to determine plasmids [38].

### 2.10. Data Availability and Accession Numbers

Scaffold sequences of *L. amylovorus* strains were deposited in the GenBank database under the accession numbers listed in Table 1.

## 3. Results

### 3.1. De Novo Genome Assembly, Average Nucleotide Identity and Roary Pangenome

In total, a de novo genome assembly was carried out on 28 genomes of *L. amylovorus* strains. The number of assembled contigs varied from 49 to 128, N50 and L50 were in a range of 36,990–173,037 bp and 4 to 16 contigs, respectively. Genome size ranged from 1.8 to 2.1 Mbp with average GC content of 37–38% (Table 2).

Calculation of ANI values was performed against a type strain genome of *L. amylovorus* DSM20531. The ANI values of all used *L. amylovorus* genomes were higher than the 95% recommended for species delineation for which reason all used genomes can be classified as *L. amylovorus* species (Table 2).

Roary successfully generated 8834 different orthologous groups of proteins from 45 genomes, which were subsequently separated into core genes (604; 44 ≤ strains < 45), soft-core genes (481 genes in 42 ≤ strains < 44), shell genes (1504 genes in 6 ≤ strains > 42) and cloud genes (6245 genes in <6 strains). The Roary results were used to compare CDS patterns surrounding antibiotic resistance genes.

### 3.2. MIC Profile Determination and Beta-Lactamase Activity

The MIC values of 10 different antibiotics were obtained for 28 *L. amylovorus* strains from wild boars and domestic pigs of which 15 strains showed an MIC above the established cut-off values for at least one antibiotic. The results of this study revealed that *L. amylovorus* strains from wild boars were more susceptible (12/19) to the tested antibiotics than strains from domestic pigs (1/9; Table 3 and Table 4). Resistance to ampicillin (6/9) and erythromycin (3/9) was only observed in *L. amylovorus* strains from domestic pigs. Resistance to clindamycin was observed in four out of nine domestic pigs with an MIC range from 8 mg/L to >8 mg/L in comparison with one resistant isolate with an 8 mg/L MIC range in wild boars. Resistance to tetracycline was confirmed in seven out of nine examined strains from domestic pigs with an MIC range (16 mg/L to >64 mg/L) in comparison with one resistant strain from a wild boar with an MIC (8 mg/L) only one step above the established cut-off values. On the other hand, a high level of ciprofloxacin resistance with a range from 32 to >128 mg/L was observed in *L. amylovorus* strains from both domestic pigs and wild boars, which indicates intrinsic resistance to this antibiotic in *L. amylovorus*. Regarding susceptibility to chloramphenicol, the MIC values ranging from 4–8 mg/L were observed in all isolates, where the MIC value only one step above the established cut-off value (8 mg/L) was noticed in 21% (4/19) of wild boar isolates in comparison to 11% (1/9) of isolates from domestic pigs. Additionally, ampicillin-resistant strains were analyzed for beta-lactamase activity using a nitrocefin test. Beta-lactamase activity was not confirmed by this test in any strain phenotypically resistant to ampicillin.

### 3.3. Detection of Antibiotic Resistance Determinants in L. amylovorus

Genomes of all *L. amylovorus* strains were screened for known acquired resistance genes. Overall, this analysis revealed only three genetic determinants of antibiotic resistance—*tetW* (six strains), *ermB* (three strains) and aminoglycoside adenyltransferase (*aadE*, one strain; Table 3 and Table 4). Antibiotic resistance genes *tetW* and *ermB* with the highest identity (*tetW*: 97–100%, *ermB*: 98–100%) were only confirmed in phenotypically resistant strains from domestic pigs with an MIC ≥ 32 and MIC > 8 mg/L, respectively. No transmissible antibiotic resistance determinants were identified in another two strains from wild boars and domestic pigs with an MIC for tetracycline above the microbiological cut-off (8 mg/L and 16 mg/L). Co-occurrence of *ermB* and *tetW* was observed in three out of six *tetW* positive strains. Further, resistance gene *aadE* involved in resistance to streptomycin was determined in only one strain from a wild boar with an MIC value of 128 mg/L. However, in comparison to *tetW* and *ermB*, a low identity of 83% was determined using the Abricate program. Based on blastn, the highest identity of 83% was shown by the sequence previously identified in many bacterial species, such as *Campylobacter coli* (GenBank: KC876751.1), *Streptococcus agalactiae* Sag153 (GenBank: CP036376.1) and *Clostridioides difficile* TW11-RT078 (GenBank: CP035499.1). A low identity of 83% was also revealed when the gene from our strain was compared with *aadE* genes from another two *L. amylovorus* strains (PMRA3 and MGYG-HGUT.00161). Similarly, nucleotide global alignment using ClustalO revealed a low 82–83% nucleotide identity with reference gene *aadE* (*Campylobacter jejuni* plasmid pCG8245, GenBank: AY701528.1) and the *aadE* gene mentioned above. Phenotypic resistance to ampicillin in domestic pigs was not explained. No *bla* genes encoding beta lactamases were detected. Regarding the presence of prophage sequences analyzed by the PHASTER web server, no prophage sequences related to antibiotic resistance genes were detected in our isolates.

### 3.4. Analysis of tetW Sequences and Their Flanking Regions

**Figure 1:**
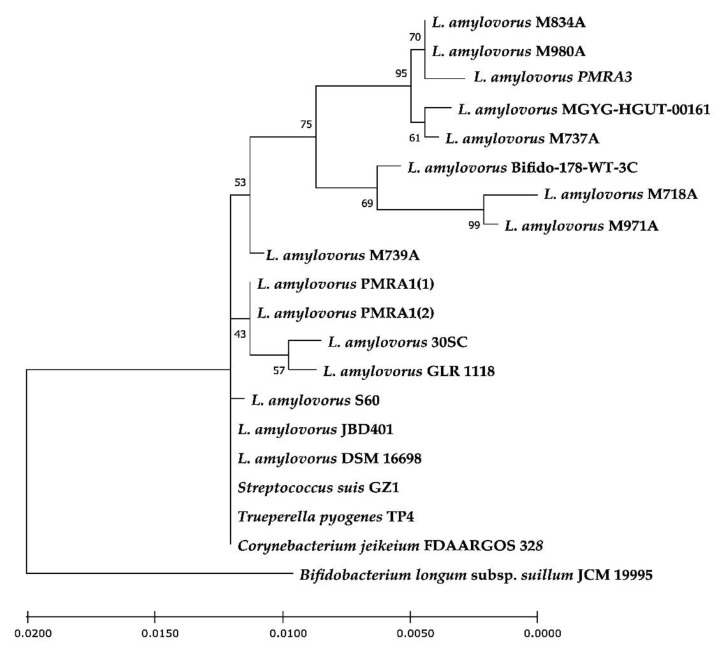
The phylogenetic tree of a *tetW* gene, showing the relationship between the *tetW* genes from *L. amylovorus* (received from our study and NCBI) and the *tetW* sequences of selected bacterial species showing the highest similarity to *L. amylovorus tetW* genes (based on the blastn analysis). The evolutionary history was inferred using the Maximum Likelihood method and the Tamura 3-parameter model [36]. Bootstrap values (1000 replicates) were applied and the percentage of trees in which the associated taxa clustered together is shown next to the branches. The tree is drawn to scale, with branch lengths measured in the number of substitutions per site. There were a total of 1932 positions in the final dataset. The phylogenetic tree was rooted with the *tetW* from *Bifidobacterium longum* subsp. *suillum* JCM 19995 as an outgroup. Evolutionary analyses were conducted in MEGA X [34].

The nucleotide sequences of the *tetW* genes and their flanking regions up to 5 kb, including CDS, were compared among *tetW* positive *L. amylovorus* strains retrieved from our study (six strains) and from the NCBI database (nine strains). The number of single nucleotide polymorphisms (SNPs) between *tetW* from studied *L. amylovorus* strains ranged from 0 to 22 (performed in the software MEGA X using the pairwise method). According to the blastn analysis, a high degree of identity in *tetW* genes was also observed between *L. amylovorus* and some pathogenic or potentially pathogenic bacterial species. *tetW* genes from *Streptococcus suis* GZ1 (GenBank: CP000837.1), *Trueperella pyogenes* TP4 (GenBank: CP033905.1) and *Corynebacterium jeikeium* FDAARGOS_328 (GenBank: CP022054.2) demonstrated sequences identical or almost identical (with the number of SNPs ranging from 0 to 3) with *L. amylovorus* strains M739A, GLR 1118, DSM 16698, JBD401, PMRA1 and S60 (Figure 1).

**Figure 2:**
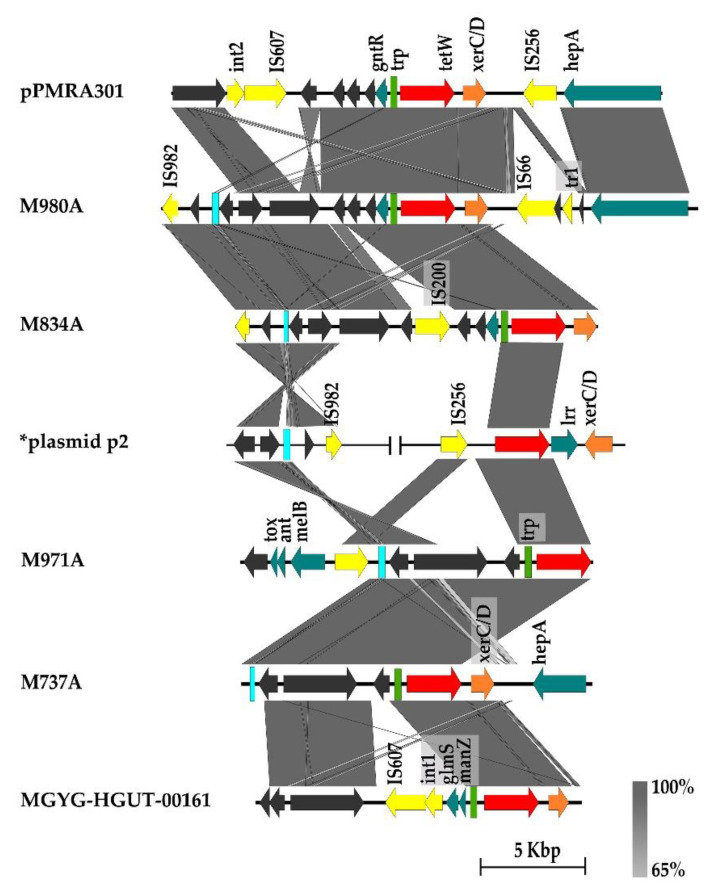
Organization of CDS in the contigs harboring *tetW* and *xerC/D* in *L. amylovorus* strains and comparison with plasmid pPMRA301 and plasmid p2 from *L. amylovorus* PMRA3 and GLR1118, respectively. Yellow arrow—CDS associated with mobility (e.g., IS—transposase, tr—putative transposase, int—putative integrase), red arrow—*tetW* gene, teal arrow—CDS with COG/PROKKA annotation, gray arrow—hypothetical protein, orange—*XerC/D* site-specific recombinase, green rectangle—*tetW* regulatory protein (*trp*), light blue—unknown misc. feature. The gray zones between sequences represent blastn sequence identity. *Plasmid p2 from GLR1118 shown only CDS identical to contigs bearing *tetW* resistance.

**Figure 3:**
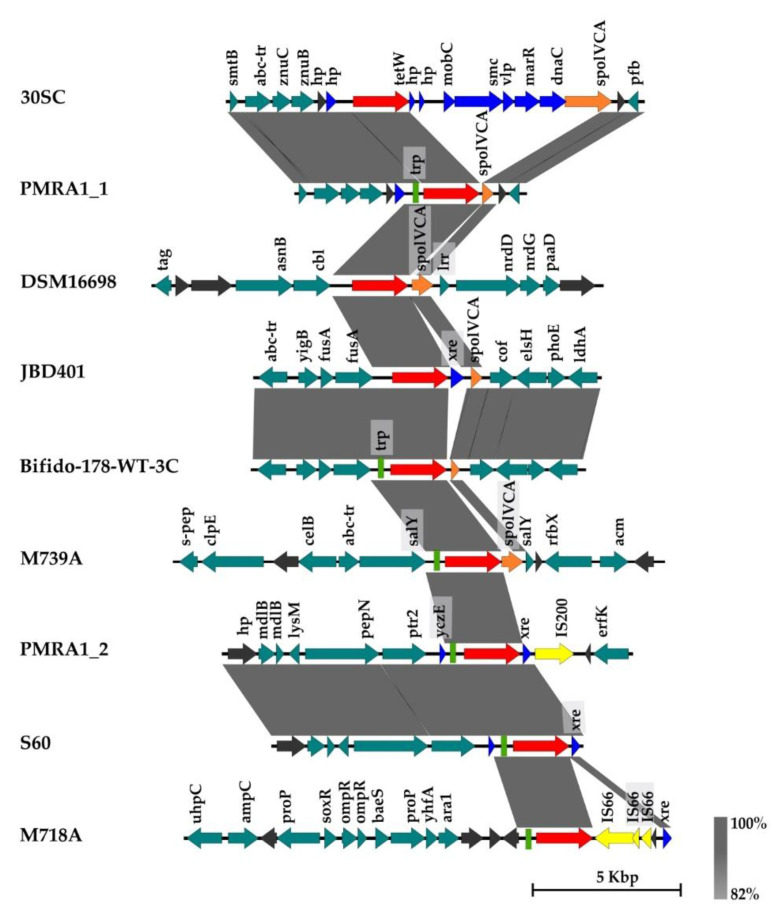
Organization of CDS in the contigs harboring *tetW* and site-specific recombinase *spoIVCA* or *tetW* and *xre* (DNA-binding transcriptional regulator) in *L. amylovorus* strains. Yellow arrow—CDS associated with mobility (e.g., IS—transposase), red arrow—*tetW* gene, teal arrow—CDS with functional annotation, gray arrow—hypothetical protein, orange—*SpoIVCA* site-specific recombinase, green rectangle—*tetW* regulatory protein (*trp*), blue arrow—CDS identified in other bacterial spp. (e.g., *Treponema succinifaciens* DSM 2489 or *Victivallales* bacterium CCUG 44730). The gray zones between sequences represent blastn sequence identity (generated by EasyFig).

Sequence analysis of CDS in the neighborhood of the *tetW* gene in *L. amylovorus* strains revealed the presence of site-specific recombinase *xerC/D*, site-specific DNA recombinase *SpoIVCA* or DNA-binding transcriptional regulator *xre*, usually directly downstream of the *tetW* gene (Figure 2 and Figure 3).

Isolates were divided in two main groups based on the presence of these CDS located in the neighborhood of the *tetW* gene. In the first group, most of the CDS located on contigs harboring gene *xerC/D* recombinase flanking *tetW* and showing similarity with CDS previously found in plasmid pPMRA301 and plasmid p2 from *L. amylovorus* PMRA3 and GLR1118, respectively. Most of these CDS coded hypothetical proteins with just an approximate function. However, some CDS coding for a variety of transposase (e.g., family transposase: IS*982*, IS*256* or IS*607*) have been frequently identified (Supplementary Data Table S1) on these contigs. Four of our isolates M737A, M834A, M971A, and M980A as well as strain MGYG-HGUT-00161 obtained from the NCBI were included in this group (Figure 2; Supplementary Data Table S1).

The second group was characterized mainly by the presence of partial or complete CDS for SpoIVCA or Xre downstream of the *tetW* gene (Figure 3). Both CDS shared high identity with sequences from other bacterial species. For instance, CDS for SpoIVCA was found in *Clostridioides difficile* (GenBank: MH229773.1), *Treponema succinifaciens* (GenBank: CP002631.1) and *Victivallales bacterium* CCUG 44730 (GenBank: CP027227.1) with a blastn sequence identity of 100% (nucleotide global alignment identity 41–92%).

CDS coding for part of SpoIVCA was found in six strains of *L. amylovorus* (M739A, PMRA1, DSM 16698, JBD401 and Bifido-178-WT-3C, 30SC). CDS coding for Xre identified in four *L. amylovorus* strains (JBD401, PMRA1, S60, M718A) exhibited 99–100% blastn identity (nucleotide global alignment identity ty 22–93%) to CDS in *Streptococcus suis* GZ1 (GenBank: CP000837.1) and *Trueperella pyogenes* TP4 (GenBank: CP033905.1).

Noticeably, *L. amylovorus* strain 30SC harbors eight CDS located downstream of the *tetW* gene, which were not identified in any *L. amylovorus* but were identified in, for example, *Treponema succinifaciens* DSM 2489 (GenBank: CP002631.1) or *Victivallales bacterium* (GenBank: CP027227.1). In some isolates, the sequence for the 14-amino-acid *tetW*-regulatory peptide (*trp*) was identified upstream of the *tetW* gene. This sequence was originally identified in *tetW*-positive *B. animalis* subsp. *lactis* strain F11 [39]. The occurrence of *trp* appears to be coincidental.

### 3.5. Analysis of the ermB Sequence and Its Flanking Regions

Five *ermB* positive strains were available for the analysis of the *ermB* gene and its surrounding region in *L. amylovorus* strains. High identity (99.32 to 100%) was observed between *ermB* genes in all five *L. amylovorus* strains with the number of SNPs ranging from zero to six. High identity of the *ermB* gene ranging from 99.86 to 99.6% (with the number of SNPs ranging from one to nine) was also shared with a sequence of *Streptococcus pneumoniae* (GenBank: MT489699), *Enterococcus faecalis* (GenBank: MK784777.1) and *Lactobacillus johnsonii* (GenBank: CP039261.1) from the NCBI.

**Figure 4:**
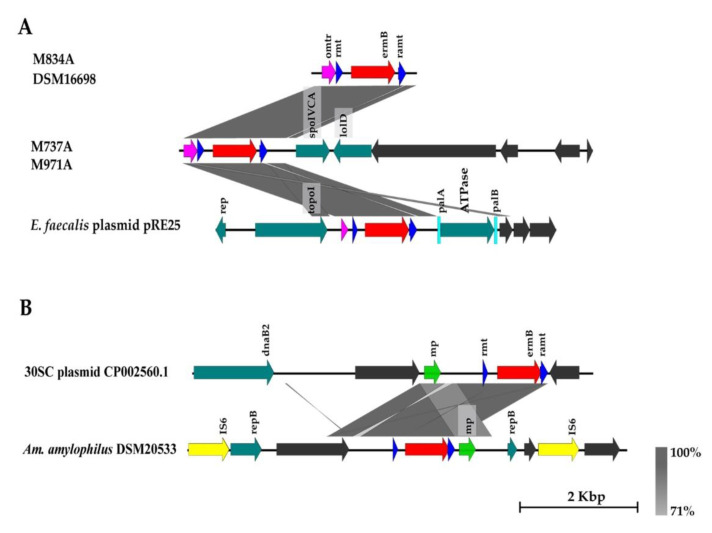
Organization of CDS in the contigs harboring *ermB* gene. (**A**) contigs bearing omega transcriptional repressor (*omtr*) near *ermB*. (**B**) CDS pattern with mobilization protein (*mp*) near the *ermB* gene. (**A**) and (**B**): dark blue arrow—23S rRNA methyl transferase (*rmt*) and rRNA adenine methyltransferase gene (*ramt*), red arrow—*ermB* gene, teal arrow—CDS with functional annotation, gray arrow—hypothetical protein, pink arrow—omega transcriptional repressor (*omtr*), light blue rectangle—palindromatic sequences, yellow arrow—CDS associated with mobility (IS—transposase). The gray zones between sequences represent blastn sequence identity.

A genetic element consisting of 23S rRNA methyl transferase (*rmt*), *ermB* and rRNA adenine methyltransferase gene (*ramt*) was determined in all *ermB* positive isolates. Additionally, in four out of five isolates, this genetic element was accompanied upstream of *ermB* and *rmt* with a sequence corresponding to the omega transcriptional repressor (*omtr*), which was previously annotated in *Streptococcus suis* ICE element ICESsuYS430 (GenBank: MK211825.1). This pattern including *omtr*, *rmt*, *ermB* and *ramt* was detected with 100% identity by blastn in many bacterial species, particularly of the genera *Enterococcus* and *Streptococcus*, e.g., *E. faecalis* plasmid pRE25 (GenBank: X92945.2) and *S. pneumoniae* Tn6822 (GenBank: MT489699.1; Figure 4A; Table S2). In comparison, the strain *L. amylovorus* 30SC harbors a sequence coding for a mobilization protein (MP) instead of *omtr*. This CDS (with 100% identity) was found downstream of *ermB* and *ramt* in *Amylolactobacillus amylophilus* DSM20533 (GenBank: CP018888.1; Figure 4B; Supplementary Data Table S2). Genes for an omega transcriptional repressor as well as a mobilization protein were mostly seen in plasmids, although carriage in chromosomes was also observed.

The *ermB* gene in *L. amylovorus* 30SC is located in the plasmid. The localization of *ermB* in plasmid in other studied strains was proven by the Platon tool in only one of our strains (M834A; Table 4). Additionally, some of the CDS in the neighborhood of *ermB* in the neighborhood of ermB in other strains have previously been found in plasmid from *L. amylovorus* and *L. crispatus* (Supplementary Data Table S2).

## 4. Discussion

Genes encoding resistance to antibiotics have been determined in intestinal bacteria from both domestic and wild animals. However, the lower abundance of antibiotic resistance genes in the resistome of wild animals as compared to food-producing animals has been observed [40].

A similar trend was also observed in our study. Only one strain with a potentially horizontally transmissible antibiotic resistance gene, specifically *aadE* which confers resistance to streptomycin, was detected in wild boars. The similarity of the gene with sequences in the NCBI was only approximately 83%, although a resistant phenotype with a high MIC (128 mg/L) was confirmed according to the microdilution method. Streptomycin is an antibiotic agent applied in food-producing animals, although it can also be produced naturally by certain strains of *Streptomyces griseus* that are commonly found in soil [41].

Increased levels of MIC for ciprofloxacin were noticed in all isolates. Formally, MIC breakpoints for ciprofloxacin are >0.5, >1 and >4 mg/L for *Enterobacterales*, *Staphylococcus* and *Enterococcus*, respectively [42]. In our study, high levels of MIC for ciprofloxacin ranging from 32 to >128 mg/L were observed in *L. amylovorus* strains, which indicates intrinsic resistance to this antibiotic in all *L. amylovorus* isolates. Generally, lactobacilli seem to be intrinsically resistant to ciprofloxacin. However, the range of MIC for *Lactobacillus* spp. for ciprofloxacin varies between 0.25 and 256 mg/L among different species [8]. The mechanism of ciprofloxacin resistance has not yet been fully clarified, since no mutations in regions of the *parC* and *gyrA* genes are generally detected in *Lactobacillus* spp. Intrinsic resistance to ciprofloxacin in lactobacilli may therefore result from cell wall structure, permeability or an efflux pump [43].The strains isolated from domestic pigs displayed higher phenotypic resistance as well as more frequent presence of genes encoding antibiotic resistance in comparison with wild boars. The *tetW* gene encoding resistance to tetracycline was present in seven out of nine analyzed strains and the *ermB* gene encoding resistance to erythromycin was determined in three strains. Similarly to Dec et al. [10], we also confirmed carriage of the *ermB* gene in all phenotypically resistant strains. However, not all strains phenotypically resistant to tetracycline carried *tet* genes. These genes were detected only in isolates with an MIC of 64 mg/L or above. Although consumption of tetracycline in food-producing animals has been decreasing in the Czech Republic in the last few years, tetracycline still represents more than 25% of total antibiotic sales [44]. Macrolides are considered critically important antimicrobials with the highest priority for human medicine by the WHO [45]. The consumption of macrolides in food-producing animals in the Czech Republic has fluctuated over the years [44]. The long-term high level of consumption of tetracycline and macrolides in domestic pigs worldwide is also reflected in some way in bacterial resistance profiles in both commensal (*E. coli*) and pathogenic bacteria [3,46,47]. Despite the limited number of studies on antibiotic susceptibility and detection of antibiotic determinants in intestinal lactobacilli from domestic pigs, some studies from Chinese and Taiwanese pig farms report the high occurrence of both tetracycline and erythromycin resistance in lactobacilli from fecal samples [46,48].

In general, *tetW* and *ermB* genes are widely distributed in many Gram-positive bacterial species from a variety of genera, such as *Bacillus*, *Bifidobacterium*, *Clostridium*, *Staphylococcus* and *Streptococcus* isolated from animals, humans or environmental samples [49,50,51,52,53]. According to our study, some *tetW* genes from *L. amylovorus* strains were closely related to *tetW* genes from other bacterial species, such as *Streptococcus suis* and *Trueperella pyogenes*, and *ermB* genes shared a high homology with sequences from, for example, *Enterococcus* and *Streptococcus* deposited in the NCBI database (https://www.ncbi.nlm.nih.gov/; accessed on 1 October 2022). The transfer of *tet* and *ermB* genes from lactobacilli to other bacterial species has previously been demonstrated in vitro [9,54]. However, Lactobacilli may not be the most important reservoir and source of *tetW*. Instead, gut microbiota isolates from the families *Lachnospiraceae* and *Ruminococcaceae* are the most likely reservoirs [12].

In the present study, the variability in CDS flanking antibiotic resistance genes in *L. amylovorus* suggests multiple independent mechanisms of *tetW* transmission (Figure 2, Figure 3 and Figure 4). In 7 out of 15 *L. amylovorus* strains, regions of *tetW* genes harbor CDS coding for genes previously found in plasmids, predominantly of *L. amylovorus* PMRA3 and GLR1118, indicating that the plasmid could participate to transmission. In most of these strains, CDS coding for the recombinase *xerC/xerD* family was located directly downstream of the *tetW* gene together with variable transposase in the neighborhood of the *tetW* gene. Noticeably, according to NCBI blastn DNA, sequences of CDS in these contigs, including ORF encoding *xerC/xerD*, were previously found only in the *Lactobacillus* group, indicating the possibility of transmission only between lactobacilli. The primary function of XerC/XerD recombinases is to resolve dimers of circular chromosomes and crossover at the specific *dif* site. They also cause resolution of multimers of plasmids and could be part of mobile genetic elements facilitating their integration into the genome [55].

On the other hand, in the rest of the strains harboring *tetW* this gene was located in a different genomic region and flanked downstream by two types of CDS previously found in different bacterial species. The putative mobile genetic element containing *spoIVCA* (ORF1984) and *tetW* has previously been described in *Bifidobacterium thermacidophilum* from domestic pigs [51]. According to our results, this genetic element was also confirmed in *L. amylovorus* strains from pigs, as well as in other sources, such as cattle waste, corn and yoghurt.

Two types of suspected genetic elements with *ermB* and CDS for omega transcriptional repressor or mobilization protein were noticed in *L. amylovorus* strains. A gene encoding a mobilization protein has previously been described upstream of the *ermB* gene in *L. amylophilus* [8] and, based on blastn, both CDS can be found in a variety of bacterial species, such as *Enterococcus* spp., *Streptococcus* spp. and *Lactobacillus* in association with the *ermB* or *lnu* gene, which highlights the possibility of interspecies transmission.

## 5. Conclusions

MIC profiles for selected antibiotics were determined in 28 *L. amylovorus* strains from wild boars and domestic pigs. Comparative WGS analysis revealed resistance determinants *tetW* and *ermB* in the majority of strains from domestic pigs, in comparison to only one strain from wild boars carrying antibiotic determinants with homology to *aadE*. These results suggest that wild pigs may be a suitable source of lactobacilli for the subsequent selection of probiotic strains, because they pose a lower risk of resistance gene transfer compared to domestic pigs. Based on the study of CDC flanking antibiotic resistance genes, it seems that there are different mechanisms of transmission of these genes, which indicates different risks of transmission, especially for the *tetW* gene. This study has helped to select *L. amylovorus* isolates with a reduced risk of antibiotic resistance gene transfer, which will be further studied for probiotic properties. The selected isolates will be added to the probiotic composition and tested in vivo in experiments on weaned piglets.

## Figures and Tables

**Table 1:** Newly sequenced genomes and NCBI genomes of *L. amylovorus*.

Origin	Strain	Country	Bioproject	NCBI Accession
GIT of Wild Boar	350A	Czech Republic	PRJNA886611	SAMN31135161
GIT of Wild Boar	352A	Czech Republic	PRJNA886611	SAMN31135162
GIT of Wild Boar	355A	Czech Republic	PRJNA886611	SAMN31135163
GIT of Wild Boar	374A	Czech Republic	PRJNA886611	SAMN31135164
GIT of Wild Boar	M356A	Czech Republic	PRJNA886611	SAMN31135165
GIT of Wild Boar	M374A	Czech Republic	PRJNA886611	SAMN31135166
GIT of Wild Boar	M388A	Czech Republic	PRJNA886611	SAMN31135167
GIT of Wild Boar	M477A	Czech Republic	PRJNA886611	SAMN31135168
GIT of Wild Boar	M490A	Czech Republic	PRJNA886611	SAMN31135169
GIT of Wild Boar	M492A	Czech Republic	PRJNA886611	SAMN31135170
GIT of Wild Boar	M581A	Czech Republic	PRJNA886611	SAMN31135171
GIT of Wild Boar	M583A	Czech Republic	PRJNA886611	SAMN31135172
GIT of Wild Boar	M597AA	Czech Republic	PRJNA886611	SAMN31135173
GIT of Wild Boar	M597B	Czech Republic	PRJNA886611	SAMN31135174
GIT of Wild Boar	M624A	Czech Republic	PRJNA886611	SAMN31135175
GIT of Wild Boar	M668A	Czech Republic	PRJNA886611	SAMN31135176
GIT of Wild Boar	M696A	Czech Republic	PRJNA886611	SAMN31135177
GIT of Wild Boar	M700A	Czech Republic	PRJNA886611	SAMN31135178
GIT of Wild Boar	M702A	Czech Republic	PRJNA886611	SAMN31135179
GIT of Domestic Pig	M718A	Czech Republic	PRJNA886611	SAMN31135180
GIT of Domestic Pig	M737A	Czech Republic	PRJNA886611	SAMN31135181
GIT of Domestic Pig	M738A	Czech Republic	PRJNA886611	SAMN31135182
GIT of Domestic Pig	M739A	Czech Republic	PRJNA886611	SAMN31135183
GIT of Domestic Pig	M834A	Czech Republic	PRJNA886611	SAMN31135184
GIT of Domestic Pig	M838B	Czech Republic	PRJNA886611	SAMN31135185
GIT of Domestic Pig	M971A	Czech Republic	PRJNA886611	SAMN31135186
GIT of Domestic Pig	M980A	Czech Republic	PRJNA886611	SAMN31135187
GIT of Domestic Pig	M1020A	Czech Republic	PRJNA886611	SAMN31135188
Bovine nasopharynx	S60	Canada	PRJNA533291	SAMN11456246
Wild boar feces	W3P1.019	Canada	PRJNA494875	SAMN10183302
Porcine ileum	GLR1118	Finland	PRJNA42079	SAMN02603307
GIT of domestic pigs	GRL1112	Finland	PRJNA42073	SAMN02603306
Faeces of Domestic Pig	Bifido-178-WT-3C	Germany	PRJNA561470	SAMN14558271
Tibetan pig GIT	MAG058	China	PRJNA647157	SAMN16927205
Tibetan pig, feces	MAG237	China	PRJNA647157	SAMN16927384
Human gut	MGYG-HGUT-00161	China	PRJEB33885	SAMEA5849662
Human feces	SRR341604-bin14	China	PRJEB37358	SAMEA7847929
Maotai-flavor liquor	MT30	China	PRJNA222257	SAMN02797775
Cattle waste-corn fermentations	DSM16698	Inner Mongolia—China	PRJNA53145	SAMN02603487
Domestic pig intestines	30SC	South Korea	PRJNA348650	SAMN05913067
Cattle waste-corn fermentation	DSM20531	South Korea	PRJNA285821	SAMN03761145
Traditional yoghurt	JBD401	South Korea	PRJNA428540	SAMN08294903
Domestic pig feces	PMRA1	South Korea	PRJNA474419	SAMN09303046
Domestic pig feces	PMRA3	South Korea	PRJNA726865	SAMN18972587
Bovine feces	1394N20	South Korea	PRJNA763780	SAMN21449406

GIT: gastrointestinal tract.

**Table 2:** Genome assembly and ANI values in *L. amylovorus* strains.

	Strain	Assembly Number	Number of Contigs	Size (bp)	L50 (Contigs)	N50 (bp)	GC (%)	ANI (%)
**Wild boars**	350A		50	1,950,158	5	120,475	38.10	96.94
	352A		68	2,089,551	6	137,004	37.92	96.73
	355A		80	1,975,531	6	131,266	37.94	96.94
	374A		59	2,100,142	5	172,046	37.92	96.89
	M356A		62	2,141,721	5	137,678	37.65	97.12
	M374A		49	1,944,827	4	173,037	38.01	97.25
	M388A		53	1,953,070	5	138,645	38.00	97.05
	M477A		94	2,062,167	10	71,381	37.83	97.03
	M490A		82	2,083,052	8	85,196	37.74	96.85
	M492A		56	1,806,111	8	93,745	38.18	97.27
	M581A		75	1,962,117	5	171,845	37.83	96.9
	M583A		64	1,976,173	6	111,868	37.87	96.84
	M597AA		126	2,098,617	12	56,554	37.78	98.79
	M597B		122	2,095,652	13	56,022	37.78	98.65
	M624A		112	1,995,845	12	57,998	37.84	98.6
	M668A		60	1,965,416	4	172,888	37.94	96.95
	M696A		126	1,950,594	16	36,990	37.95	98.76
	M700A		74	2,040,282	7	90,886	37.78	97.21
	M702A		57	1,933,350	6	139,432	37.96	96.96
**Domestic pigs**	M718A		59	1,820,933	6	109,032	37.96	97.17
	M737A		128	1,922,035	10	65,194	37.90	97.26
	M738A		70	1,851,242	5	92,554	38.07	97.14
	M739A		87	1,873,017	8	72,862	38.00	97.18
	M834A		128	2,037,562	13	54,720	37.8	97.29
	M838B		59	1,860,275	6	112,110	38.21	97.07
	M971A		95	1,931,587	7	118,773	38.00	97.1
	M980A		118	1,920,806	12	48,716	38.00	97.19
	M1020A		76	1,957,447	8	78,955	37.95	97.11
**NCBI database**	S60	GCA_005049155.1	74	2,004,240	15	48,176	37.90	96.93
	W3P1.019	GCA_004552585.1	180	1,872,704	29	21,308	38.30	97.35
	GLR1118	GCA_000194115.1	3	1,977,087	-	-	37.99	97.05
	GRL1112	GCA_000182855.2	4	2,126,664	1	2,036,842	38.08	97.13
	Bifido-178-WT-3C	GCA_012843555.1	110	1,935,156	17	37,403	37.90	97.04
	MAG058	GCA_016293325.1	217	2,741,244	33	25,918	38.50	98.68
	MAG237	GCA_016295345.1	271	1,640,195	61	7287	38.40	97.47
	MGYG-HGUT-00161	GCA_902363955.1	157	2,049,854	24	30,444	37.80	97.24
	SRR341604-bin14	GCA_905211795.1	52	1,959,419	7	74,890	37.80	97.37
	MT30	GCA_020149995.1	1	1,925,613	-	-	38.10	98.31
	DSM 16698	GCA_001437365.1	200	2,001,630	20	32,525	37.90	96.97
	30SC	GCA_000191545.1	3	2,097,766	-	-	38.08	97.06
	**DSM 20531**	**GCA_002706375.1**	**1**	**2,172,769**	**-**	**-**	**37.80**	**100.00**
	JBD401	GCA_002950865.1	1	1,946,267	-	-	38.20	96.74
	PMRA1	GCA_004307475.1	32	1,706,975	6	88,760	38.20	96.91
	PMRA3	GCA_006384175.1	2	2,145,019	1	2,060,784	38.11	96.95
	1394N20	GCA_021398395.1	1	2,176,326	-	-	37.80	98.19

**Table 3:** Distribution of minimal inhibition concentration (MIC) in *L. amylovorus* derived from wild boars and domestic pigs.

Antibiotics Range (mg/L)	Animal Sources	No.	MIC Values (mg/L)																
			0.063	0.125	0.25	0.5	1	2	4	8	>8	16	>16	32	64	>64	128	>128	256
Ampicillin (0.125–16)	wild boar	19				18	1												
	domestic pig	9				1	2			3		2	1						
Streptomycin (2–256)	wild boar	19							1	13		3		1			1 ^aadE^		
	domestic pig	9								5		3		1					
Tetracycline (0.5–64)	wild boar	19					1	7	10	1									
	domestic pig	9						1	1			1		1 ^tetW^	2 ^tetW^	3 ^tetW^			
Erythromycin (0.063–8)	wild boar	19	1	4	10	1	3												
	domestic pig	9		1	4	1					3 ^ermB^								
Clindamycin (0.063–8)	wild boar	19	3	1		3	7	3	1	1									
	domestic pig	9		1	3	1				1	3								
Vancomycin (0.25–32)	wild boar	19				14	5												
	domestic pig	9				8	1												
Chloramphenicol (0.25–32)	wild boar	19							15	4									
	domestic pig	9							8	1									
Kanamycin (16–2050)	wild boar	19										2		11	5		1		
	domestic pig	9												4	3		2		
Gentamicin (0.125–512)	wild boar	19				1	1	4		11		2							
	domestic pig	9			1	1			3	3		1							
Ciprofloxacin (0.125–128)	wild boar	19													7		10	2	
	domestic pig	9												1	2		5	1	

Gray zones represent values higher than the cut-off values for *L. acidophilus* according to the guidance on the characterization of microorganisms used as feed additives or as production organisms (2018). The cut-off value of Ciprofloxacin is not known. *tetW*, *ermB* and *aadE* genes have been detected by WGS analysis.

**Table 4:** Antibiotic resistance in individual *L. amylovorus* strains and association with plasmid determination.

	Strains	>Cut-Off Values	ATB Resistance Genes (Abricate)	Resistance on Plasmid	Contigs Determined as Plasmid
**Wild boars**	350A	0	0	-	4
	352A	0	0	-	0
	355A	0	0	-	1
	374A	0	0	-	1
	M668A	KAN	0	-	5
	M700A	0	0	-	1
	M702A	0	0	-	2
	M356A	CMP	0	-	2
	M374A	CMP	0	-	2
	M388A	0	0	-	1
	M624A	TET-CMP	0	-	2
	M597AA	0	0	-	1
	M597B	0	0	-	1
	M477A	STR	0	-	2
	M490A	0	0	-	3
	M492A	STR	*aadE*	NO	0
	M696A	CLI-CMP	0	-	1
	M581A	0	0	-	3
	M583A	0	0	-	2
**Domestic pigs**	M1020A	AMP-CLI	0	-	0
	M737A	AMP-TET-ERY-CLI	*ermB, tetW*	NO	2
	M738A	AMP-STR-TET	0	-	0
	M971A	AMP-TET-ERY-CLI	*ermB, tetW*	*NO*	0
	M739A	TET	*tetW*	NO	0
	M718A	STR-TET-CMP	*tetW*	*NO*	6
	M834A	AMP-TET-ERY-CLI-KAN	*ermB*, *tetW*	yes (ermB), NO	4
	M980A	AMP-TET	*tetW*	NO	4
	M838B	0	0	-	2

KAN: kanamycin, CMP: chloramphenicol, TET: tetracycline, STR: streptomycin, CLI: clindamycin, ERY: erythromycin, AMP: ampicillin.

## Data Availability

The sequenced genomes have been deposited in the NCBI database under Bioproject accession number PRJNA886611. All the remaining data supporting the findings of this study are available within the article and/or Supplementary Materials.

## Supplementary Materials

The following supporting information can be downloaded at: https://www.mdpi.com/article/10.3390/microorganisms11010103/s1, Table S1: ROARY pangenome analysis—CDS surrounding *tetW* gene; Table S2: ROARY pangenome analysis—CDS surrounding *ermB* gene.
